# Whole Brain and Cranial Size Adjustments in Volumetric Brain Analyses of Sex- and Age-Related Trends

**DOI:** 10.3389/fnins.2020.00278

**Published:** 2020-04-03

**Authors:** Marek Kijonka, Damian Borys, Krzysztof Psiuk-Maksymowicz, Kamil Gorczewski, Piotr Wojcieszek, Bartosz Kossowski, Artur Marchewka, Andrzej Swierniak, Maria Sokol, Barbara Bobek-Billewicz

**Affiliations:** ^1^Department of Medical Physics, Maria Sklodowska-Curie National Research Institute of Oncology Gliwice Branch, Gliwice, Poland; ^2^Department of Systems Biology and Engineering, Silesian University of Technology, Gliwice, Poland; ^3^Biotechnology Centre, Silesian University of Technology, Gliwice, Poland; ^4^Brachytherapy Department, Maria Sklodowska-Curie National Research Institute of Oncology Gliwice Branch, Gliwice, Poland; ^5^Laboratory of Brain Imaging, Nencki Institute of Experimental Biology of Polish Academy of Sciences, Warsaw, Poland; ^6^Department of Radiology, Maria Sklodowska-Curie National Research Institute of Oncology Gliwice Branch, Gliwice, Poland

**Keywords:** MRI, voxel based segmentation, volumetry analysis, sex studies, aging

## Abstract

Our goal was to determine the influence of sex, age and the head/brain size on the compartmental brain volumes in the radiologically verified healthy population (96 subjects; 54 women and 42 men) from the Upper Silesia region in Poland. The MRI examinations were done using 3T Philips Achieva with the same T1-weighted and T2-weighted protocols. The image segmentation procedures were performed with SPM (Statistical Parameter Mapping) and FSL-FIRST software. The volumes of 14 subcortical structures for the left and right hemispheres and 4 overall volumes were calculated. The General Linear Models (GLM) analysis was used with and without the Total Brain Volume (TBV) and Intracranial Volume (ICV) parameters as the covariates to study the regional vs. global brain atrophy. After the ICV/TBV adjustments, the majority of sex differences in the specific volumes of interest (VOIs) revealed to be linked to the difference in the head/brain size parameters. The analysis also confirmed the significant effect of the aging process on the brain loss. After the TBV adjustment, the age- and sex-related volumetric trends for the gray and white matter volumes were observed: the negative age dependence of the gray matter volume is more pronounced in the males, while in case of the white matter the positive age-related trend in the female group is weaker. The local losses of the left caudate nucleus and the right thalamus are more advanced than the global brain atrophy. Different head-size correction strategies are not interchangeable and may yield various volumetric results, but when used together, facilitate studies on the regional dependencies inherent to a healthy, but aging, brain.

## 1. Introduction

Advanced imaging methods development, such as magnetic resonance imaging (MRI), allows for brain exploration. Before MRI, post-mortem examinations were the only approach to studying the differences in brain structural characteristics. The early application of MRI focused on the volumetric analysis of the whole-brain cortical gray matter. Nowadays, there is a new approach using the anatomical MRI reference images combined into templates. These digital templates reveal the differences in environmental, phenotypic, genetic and developmental factors, overall brain features such as brain shape, size and volume vary across different populations (Gogtay and Thompson, 2010; Sivaswamy et al., 2019). The developmental studies employing volumetric whole-brain methods showed that the overall total brain volume follows a curvilinear, inverted U-shaped pattern of growth from birth to adolescence when it starts to decrease considerably (Giedd et al., 1999; Tang et al., 2010). The pathological injuries accumulate in the regions of high vulnerability; however, their extent varies amid brain regions (Skorupa et al., 2017). The aging brain undergoes biochemical, molecular, structural and functional changes. However, there is still a debate on the role of sex: the sex brain differences are often claimed to exist (Lehtola et al., 2019) and to have biological and evolutionary roots (Trollor and Valenzuela, 2001; Cahill et al., 2004). On the other hand, some researchers question the sexual dimorphism—they claim that the individual brain is rather intersexual and comprises a mosaic of features of more or less male/female character (McCarthy, 2016). Others argue that sex plays a minor role in the neuroanatomical volume differences, and most differences are related to the intracranial brain volume (ICV) (Pintzka et al., 2015). Still, others think that it is not the increase in the size of human brains alone, but mainly the specialization of the cortical circuits that appears to be critical. The human cerebral cortex architecture, unique in many aspects, makes the brain morphological differences less significant (Defelipe, 2011). The shape and size of human brains may also vary across the racial groups, as reveals from the comparisons of the MRI templates obtained for various populations (Xie et al., 2015; Rao et al., 2017). Unfortunately, there are many additional factors, not related to demographic aspects or environmental factors, that may affect the volumetric brain patterns, for instance, the technical details of the MRI acquisition, the choice of the adjustment and statistical methods or the applied transformations (Clark et al., 2006; Han et al., 2006; Allen et al., 2008; Mandal et al., 2012; Ruigrok et al., 2014; Velasco-Annis et al., 2018). Inconsistency of the reported morphometric findings may, thus, reflect the variable extent to which the above methodological issues vary regarding sample characteristics (e.g., age, number, and ethnicity of the subjects)—the cross-sectional character of the study, MRI hardware (e.g., field strength, coils), scan protocols (e.g., resolution and contrast), and image analysis approach (e.g., manual/automated MRI segmentation). All of this yield a significant challenge for comparing the brain structures and functions in neuroscience research (Joel et al., 2015). In volumetric studies of various neurological pathologies or the aging brain, two main whole-brain normalizations are used: to the total brain volume (TBV) or the intracranial volume (ICV). However, there is no consensus on the most accurate way of the brain/head-size adjustment in statistical analyses (Arndt et al., 1991; Mathalon et al., 1993; O'Brien et al., 2006). Since TBV and ICV have their advantages and disadvantages (John et al., 2015), we decided to compare the regional brain volumes using both measures. The interactions and associations were studied in the GLM (General Linear Model) ANCOVA environment with TBV, ICV, age, sex as the co-variates and the sex-age interaction. The adjustments to TBV or ICV revealed to be useful indicators of brain atrophy in diabetes (Hirabayashi et al., 2016), thus, in this paper, we aimed to describe the volumetric regional brain differences on the background of the global brain atrophy in a radiologically verified (by an experienced radiologist) and neurologically healthy Polish population.

## 2. Materials and Methods

### 2.1. Human Subjects

The study sample was drawn from a database of 100 volunteers selected as the control group (homogeneous in terms of its ethnicity) from the area of Upper Silesia region in Poland. The criteria of inclusion to the studied sub-population involved the age above 18 and good health status, i.e., the absence of acute or chronic diseases (no neurological disorders or surgical history). All the participants underwent full brain MRI examinations in Radiology and Diagnostic Imaging Department of Maria Sklodowska-Curie National Research Institute of Oncology Gliwice branch between 2013 and 2014. It is essential to underline that the obtained images were validated for the lack of pathology by the experienced and always the same radiologist. Four subjects were excluded from the investigation due to the presence of silent gross brain lesions (3 cases) and due to the image artifacts (1 case), resulting in a final sample of 96 subjects (42 males and 54 females, aged from 20 to 66 years; median age 37.0 years, 25th percentile 29.0 years, 75th percentile 50.0 years). Though the subjects were randomly chosen among the Polish population, the age and sex distributions were found to reflect the characteristics of the whole population, as reveals from the Statistical Atlas of Slaskie Voivodeship edited by Central Statistical Office of Poland (https://stat.gov.pl/en/topics/other-studies/cities-voivodship/). To reflect the age distribution in the demographic description, the subjects were divided into three age groups: 20–30 years, 31–40 years, and 41–66 years and additionally grouped according to the sex. The appropriate groups are presented in Table 1. This table is only informative, as the calculations were performed using the General Linear Models (GLM) analysis from Statistica software (see Statistical Analysis below for the details).

**Table 1:** Demographic characteristics of the individuals.

	**Total sample (*n* = 96)**	**Males (*n* = 42)**	**Females (*n* = 54)**	***p***
Median age (*Q*_1_-*Q*_3_)	37.0 (29.0–50.0)	35.5 (29.0–43.0)	38.5 (29.0–50.0)	0.209^a^
Age distribution of subjects (%)				
20–30	*n* = 31 (32.3%)	*n* = 15 (35.7%)	*n* = 16 (29.6%)	
31–40	*n* = 28 (29.2%)	*n* = 15 (35.7%)	*n* = 13 (24.1%)	
41–66	*n* = 37 (38.5%)	*n* = 12 (28.6%)	*n* = 25 (46.3%)	

Q_1_-lower quartile, 25th percentile.

Q_3_-upper quartile, 75th percentile.

a *UMann-Whitney test*.

The age distributions in the studied groups were right skewed (non-symmetric). The males and females sub-groups are of a similar median age (Table 1; *p* = 0.209).

### 2.2. Data Acquisition

A T1-weighted scan was performed on Philips Achieva 3T system (Radiology and Diagnostic Imaging Department in Maria Sklodowska-Curie National Research Institute of Oncology, Gliwice Branch, Poland). A 3D spoiled gradient echo sequence was used (T1-FFE) with TE = 2.9 ms, TR = 20 ms and flip angle of 20° and parallel imaging techniques SENSE. The acquisition matrix was 256 × 256 in the x and y dimensions yielding a voxel dimension of 1 × 1 mm. The spacing between the slices was 1 mm, and the slice thickness was 2 mm. A T2-weighted scan was performed using 2D turbo spin-echo technique with TE = 80 ms and parallel imaging techniques SENSE. TR varied, depending on the number of slices, but always was longer than 2,500 ms. The acquisition matrix was adjusted to the brain size; however, the voxel size of 1 × 1 × 1 mm was always maintained. An experienced certified radiologist evaluated all MR images to identify the image artifacts and to exclude the presence of morphological pathologies (silent gross brain lesions). The original MR images encoded in DICOM were converted to the NIfTI format used by FSL, SPM, MRIcron and many other brain imaging tools using DCM2NII (http://people.cas.sc.edu/rorden/mricron/dcm2nii.html).

### 2.3. Image Processing

The images were pre-processed to increase the quality and performance of the applied methods. The MR images were denoised using the publicly available MRI Analysis Software: FSL's SUSAN 3D noise reduction tool (Smith and Brady, 1997). The DICOM data files were processed in full 3D mode taking into account the brightness threshold differences separately for each image and each volunteer. The image filtering procedure was performed by the SNR (signal-to-noise ratio) and CNR (contrast-to-noise ratio) values determination. The brightness threshold was optimized to be higher than the noise level and less than the contrast of the underlying image. The Gaussian mask was set to a default size of 3 × 3 × 3 voxels.

### 2.4. Image Segmentation

#### 2.4.1. Segmentation of Cerebral Spin Fluid, White and Gray Matter

The unified segmentation procedure implemented in Statistical Parameter Mapping (SPM) software (Wellcome Department, University College, London, UK) was applied (Ashburner and Friston, 2005). To improve the quality of voxel classification (especially in the cerebrospinal fluid determination), two-channel T1-WI and T2-WI segmentations were used. The bias corrections applied in our study were as follows: the full width at half maximum (FWHM) of 60 millimeters cutoff and the bias regularization of 0.0001. Due to the partial volume effect, the number of Gaussians representing the intensity distribution for each tissue class was optimized: two Gaussians were used for gray matter (GM), two for white matter (WM), two for cerebrospinal fluid (CSF), and four for the remaining classes. The clean parameter was set as light. Each voxel was assigned a probability of belonging to a particular tissue class based on its intensity and information from the prior probability images. The classified voxels were automatically and visually checked and saved in the native space of the original images. The probability images contain the values in the range from 0 to 1, representing the prior probability of a voxel being either GM, WM or CSF.

#### 2.4.2. Segmentation of Subcortical Structures

The voxels classified in the subcortical structures were segmented automatically using FIRST (Patenaude et al., 2011). The algorithm transforms the MR images using the 12 degrees of freedom linear fit transformations to match the orientation of the MNI152 standard template image, and then segment the structures using the shape-based method (Patenaude et al., 2011; Perlaki et al., 2017). The registrations were visually checked for each subject. Due to the reported errors in the brain stem segmentation (Velasco-Annis et al., 2018), not all individual structures and parcels were analyzed for this report. The following left (L) and right (R) subcortical structures were chosen to determine the volumetric parameters: hippocampus (HIP), putamen (PUT), thalamus (THA), caudate (CAU), pallidum (PAL), amygdala (AMY) and the accumbens area (ACC). The calculations were performed with FSL-FIRST tool (FSL's build: 507) initiated by the *run*_*first*_*all* script using the default settings. The technical details of the FIRST algorithm were described previously (Patenaude, 2007; Patenaude et al., 2011). The segmentation results were analyzed in a native space of the original T1-weighted image. For the boundary correction, the auto option was chosen, which is the default behavior of the *run*_*first*_*all* script. Finally, the successful segmentations were visually verified, and their masks were extracted into the separate files from the single image containing the labels of 14 segmented subcortical structures (*output_name_all_fast_firstseg.nii.gz*).

### 2.5. Determination of Volumetric Data

Each step of the image processing pipeline was evaluated qualitatively. Due to the 1 mm x 1 mm x 1 mm isovoxel size, the volumetric data from the SPM segmentation was determined by counting the probability in the segmented voxels for the appropriate classes (GM, WM, CSF) of the brain. The Total Brain Volume (TBV) was calculated as a sum of the gray and white matter volumes. The Intracranial Volume (ICV) was calculated as a sum of the TBV and the cerebrospinal fluid volumes. The volumetric parameters of the subcortical structures were determined by the summation of the FSL segmented isovoxels for each subcortical class. The resulting data were calculated in absolute units (mL).

### 2.6. Statistical Analysis

In order to determine the brain volumetric features related to age and sex, the moderated General Linear Models (GLM) analysis with interaction term (O'Brien et al., 2006, 2011; Kim, 2018) was done using Statistica software. The adjustment for the head size was performed at the group level, and the TBV and ICV parameters were included as covariates in the statistical analysis (O'Brien et al., 2006). The residuals from the fitted models (the predicted and residual scores) were analyzed to find the outliers. Moreover, the homogeneity of variance (Levene test) and normality of the distribution of residuals (Shapiro–Wilk test) were also checked in the studied subgroups (Miller and Haden, 2006). The models with sigma-restricted parametrization for 17 volumetric classes were created. In the first stage of the statistical analysis, the unadjusted (for the head size) GLM models with two independent explanatory variables—sex and age, including an interaction term, were created. Then, the extra models were also performed to explain the TBV and ICV variability (section 3.2). In the last stage, to reveal and compare the regional brain atrophy for the anatomical regions the ICV adjusted (additional information—see section 3.3) and TBV adjusted (additional information—see section 3.4) two independent moderated GLM analyses were conducted (Hirabayashi et al., 2016). The estimation was verified for the overall statistical significance of the models. Moreover, the standardized regression coefficients (β) and the significance of the regressor effects (*p*-values) were calculated to characterize age-related dimorphism and in the subsequent models, to adjust the TBV and ICV factors in this description. The *p*-values less than 0.05—a predetermined significance level—were accepted as indicating that the observed result would be highly unlikely under the null hypothesis (Király et al., 2016; Wang et al., 2019).

Bonferroni correction was used for the multiple comparisons (Armstrong, 2014). To optimally balance between Type I and Type II error, we took the correlation between the dependent variables (the volumes of the seventeen structures) into account by using the Simple Interactive Statistical Analysis Bonferroni tool. Using a Bonferroni correction that treats the variables as independent (proper Bonferroni: alpha/number of tests) would lead to a too stringent correction, as the dependent variables are not obtained in independent sub-groups (Wang et al., 2019). The subcortical volumes showed a mean correlation coefficient of *r* = 0.565, leading to an equivalent corrected alpha of 0.0146.

**Figure 1:**
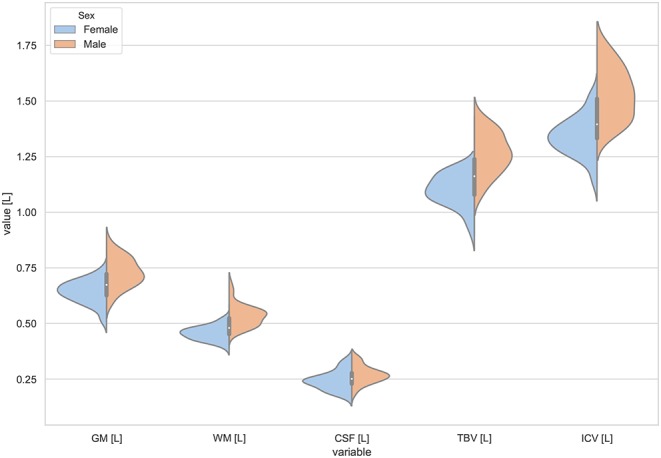
The violin plot of the volumes distribution for the gray matter (GM), white matter (WM), cerebrospinal fluid (CSF), total brain volume (TBV), and the intracranial volume (ICV) in females and males.

**Figure 2:**
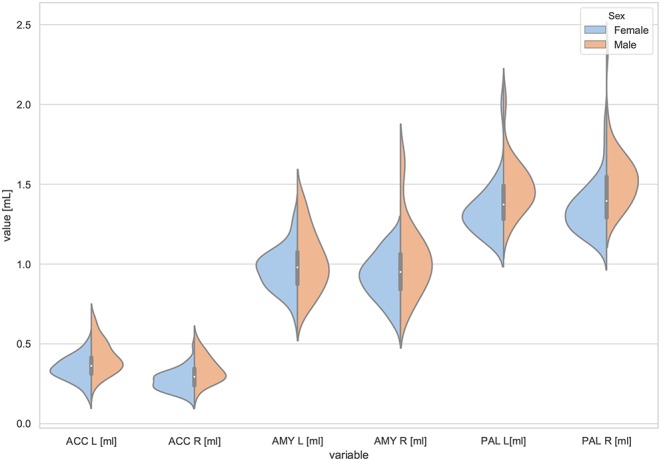
The violin plot of the volumes distribution of the left and right accumbens areas (ACC L, ACC R), left and right amygdala (AMY L, AMY R), and the left and right globus pallidus (PAL L, PAL R) in females and males.

**Figure 3:**
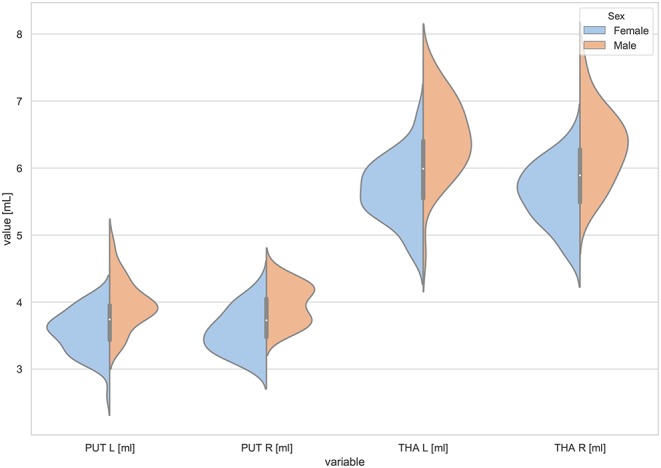
The violin plot of the volumes distribution for the left and right putamens (PUT L, PUT R) and the left and right thalamus (THA L, THA R) in females and males.

**Figure 4:**
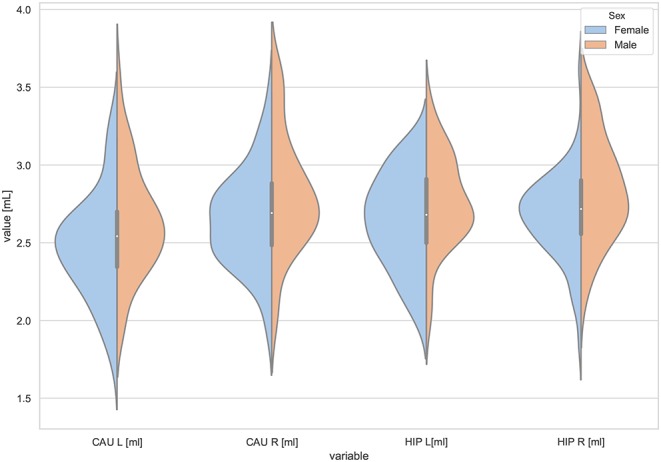
The violin plot of the volumes distribution of the left and right caudate nuclei (CAU L, CAU R) and the left and right hippocampus (HIP L, HIP R) in females and males.

**Figure 5:**
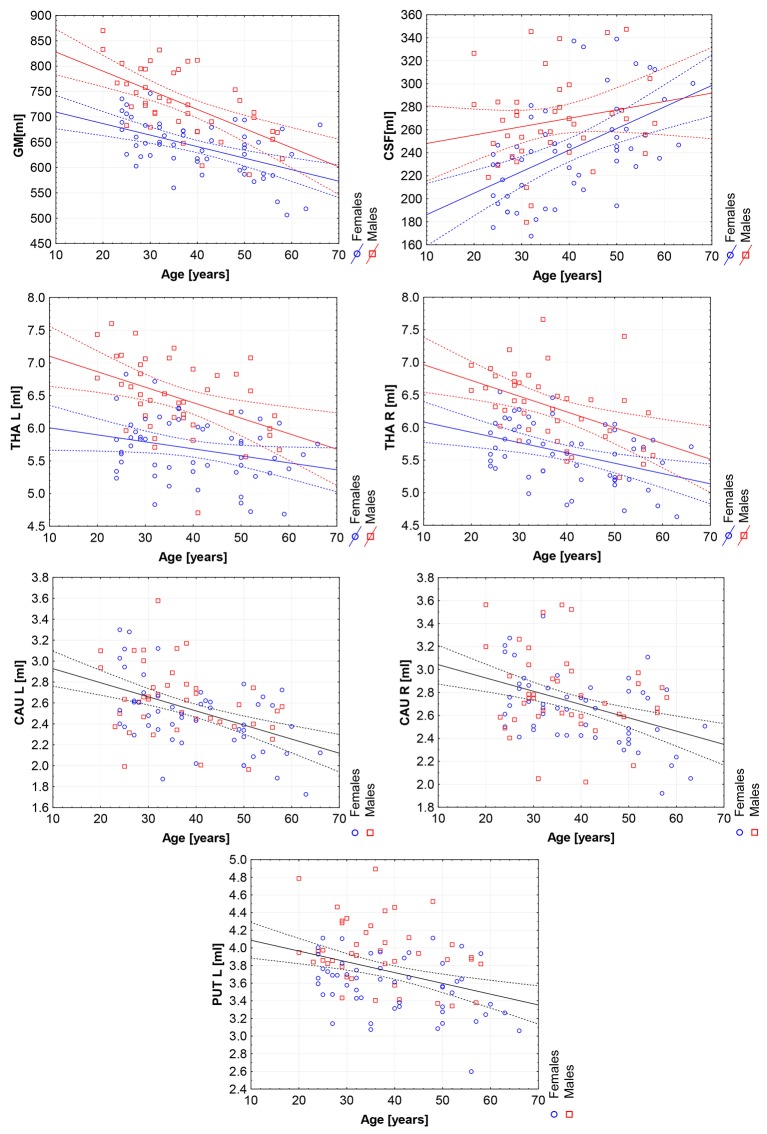
The scatter plots with trend lines and 95% confidence intervals for the age dependent brain structures: the gray matter (GM), cerebrospinal fluid (CSF), left thalamus (THA L), right thalamus (THA R), left caudate nucleus (CAU L), right caudate nucleus (CAU R), and the left putamen (PUT L).

**Figure 6:**
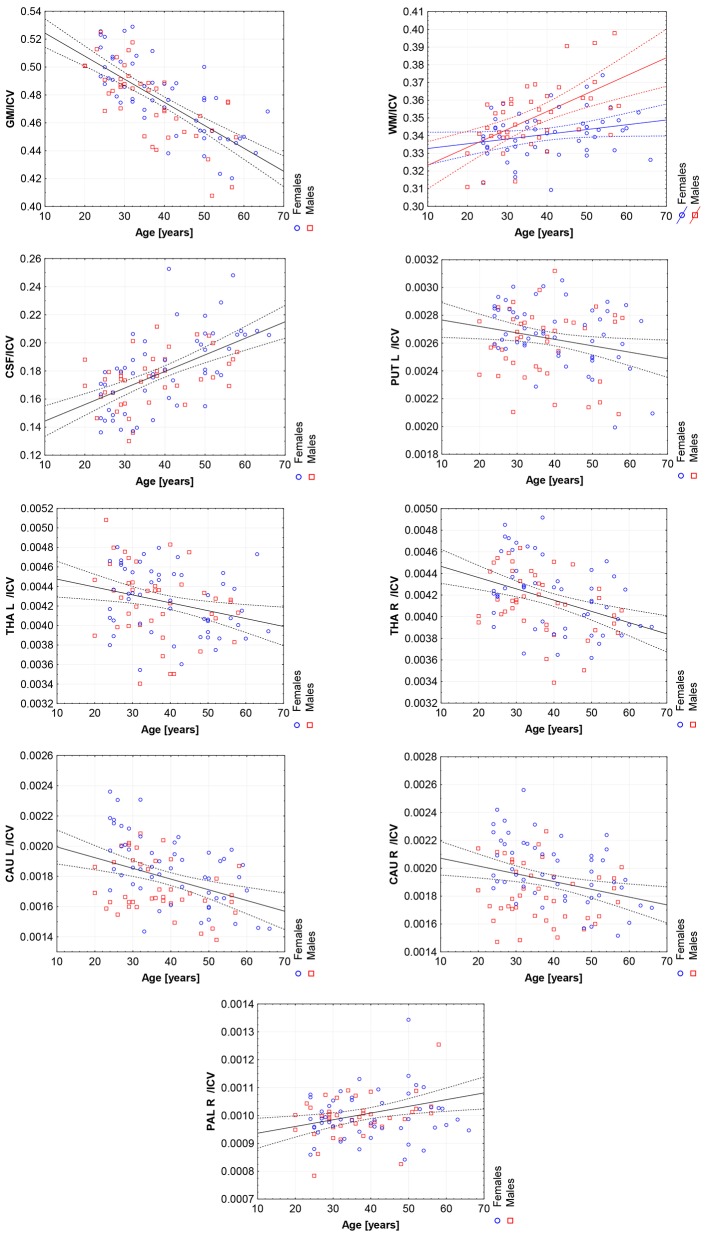
The scatter plots with the trend lines and 95% confidence intervals for the age dependent brain structures normalized with ICV: the gray matter (GM), white matter (WM), cerebrospinal fluid (CSF), left putamen (PUT L), left thalamus (THA L), right thalamus (THA R), left caudate nucleus (CAU L), right caudate nucleus (CAU R), and right pallidum (PAL R).

**Figure 7:**
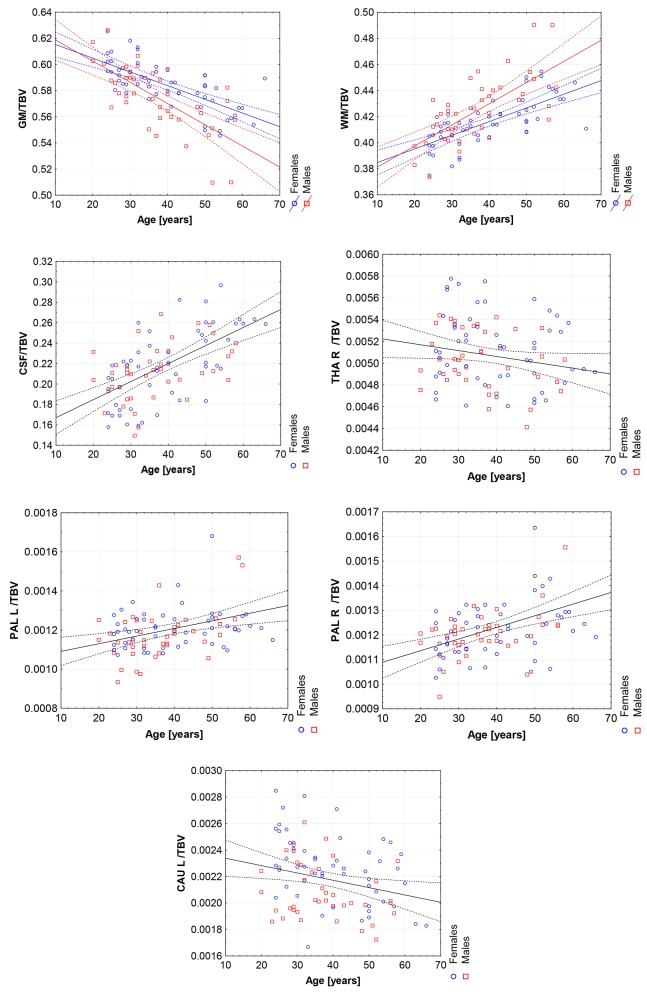
The scatter plots with the trend lines and 95% confidence intervals for the age dependent brain structures normalized with TBV: the gray matter (GM), white matter (WM), cerebrospinal fluid (CSF), right thalamus (THA R), right pallidum (PAL R), left pallidum (PAL L), and left caudate nucleus (CAU L).

Moreover, the violin plots (Figures 1–4) and the scatter plots with regression lines and 95% confidence intervals (Figures 5–7) were also presented to visualizes the obtained results. The violin plots estimate the data distribution by using a kernel density function (Weissgerber et al., 2017).

## 3. Results

The volumes of 14 subcortical structures for the left (L) and right (R) hemispheres and 4 overall volumes were calculated. The obtained volumetric parameters were subjected to the GLM analysis.

### 3.1. Unadjusted GLM Analysis

First, the sex and age differences in the cortical and subcortical brain volumes were analyzed without the head size adjustment. The results of the GLM analysis with the sex-age interaction term are presented in Table 2. Table 2 presents also the median volumes in the male and female sub-populations and the β—the standardized coefficients or weights assigned to the predictor variables. Thus, the β coefficients allow to compare the relative contributions of the independent variables in the prediction of the dependent variables. The positive/negative sign of the β coefficient is interpreted in terms of the increase/decrease of the outcome variable (Miller and Haden, 2006).

**Table 2:** The General Linear Models (with sigma-restricted parametrization) results with the two (age, sex) predictors and age-sex interaction.

	**Medians [mL]**		**Age predictor**		**Sex predictor**		**Age × Sex interaction**		**Full model**	
**GLM Models**	**Males**	**Females**	β^*^	***p*-value**	β^**^	***p*-value**	β^***^	***p*-value**	***p*-value**
GM	722.9	645.3	-0.4834	<0.001	0.919	<0.001	-0.4153	0.0974	<0.001
WM	539.6	456.1	0.1228	0.1092	0.5496	0.0365	0.1848	0.4827	<0.001
CSF	267.0	241.2	0.3517	<0.001	0.8535	0.0093	-0.5335	0.1044	<0.001
THA L	6.471	5.745	-0.3032	<0.001	0.9303	0.0012	-0.3956	0.1632	<0.001
THA R	6.360	5.637	-0.3775	<0.001	0.7835	0.0049	-0.2728	0.3242	<0.001
PUT L	3.925	3.602	-0.2836	0.002	0.3996	0.1921	0.0607	0.8442	<0.001
PUT R	3.974	3.553	-0.1571	0.0847	0.3332	0.2815	0.185	0.554	<0.001
CAU L	2.617	2.488	-0.4143	<0.001	-0.098	0.7647	0.2525	0.4467	<0.001
CAU R	2.749	2.663	-0.3525	<0.001	-0.1396	0.6783	0.3048	0.372	<0.001
HIP L	2.698	2.667	-0.0279	0.794	0.3766	0.3035	-0.1872	0.6126	0.25
HIP R	2.749	2.703	-0.1565	0.1329	0.4955	0.1628	-0.2454	0.493	0.0172
AMY L	0.983	0.979	0.0178	0.8686	0.5431	0.1414	-0.4372	0.2413	0.3927
AMY R	1.001	0.925	-0.07	0.5022	0.639	0.0749	-0.3867	0.2845	0.0316
PAL L	1.448	1.308	0.1225	0.2206	0.0631	0.8527	0.3605	0.296	<0.001
PAL R	1.539	1.333	0.1829	0.0535	0.272	0.3959	0.25	0.4408	<0.001
ACC L	0.398	0.341	-0.2137	0.0305	0.2448	0.4623	0.1225	0.7161	<0.001
ACC R	0.324	0.260	-0.1926	0.0467	0.1798	0.5824	0.2316	0.4845	<0.001

The results marked in red are statistically significant with p < 0.0146.

* The sign (+/-) of β for the age predictor - means the increasing/decreasing brain structure volume with age.

** The sign (+/-) of β for the sex predictor - means the higher/lower brain structure volumes for the males.

*** The sign (+/-) of β for the age-sex interaction - means the increasing/decreasing male/female brain.

*structure volume ratios with age*.

The violin plots of the distributions of the overall and regional brain volumes are shown in Figures 1–4. The scatter plots with regression lines, and 95% confidence intervals (Figure 5) are presented for the volumes of interest (VOIs) with statistically significant models (Table 2). When the sex differences or interactions were observed (Table 2), the trend lines and the confidence intervals were separated by sex in the scatter plots (Figure 5).

The statistically significant models were found in almost all segmented structures, except for the hippocampus and amygdala (full model *p* > 0.0146 in Table 2). Whereas for both pallidi and accumbens nuclei more stringent corrected alpha level for their regressor effects (*p*-values) has not been reached. In all models, the age-sex interactions were statistically insignificant. However, for four regions (GM, CSF and both thalami) the sex predictors were significant. All standardized sex regression coefficients indicate that the male segmented brain volumes are larger than those for women (Table 2 and Figures 1–4). The positive β values indicate the higher values for males, and the negative ones indicate the higher values for females. Such differences (as shifts of the entire distributions) are also observed in the violin plots (Figures 1, 3). The age regressor effects were significant for seven VOIs: gray matter, CSF, both thalami, both caudate nuclei and left putamen. The regression coefficients for these dependencies show a negative correlation with age, except the volume of CSF (the CSF volume increases with age). Such relationships are also seen in the scatter plots of the obtained volumes, along with the sex effects for GM, CSF and both thalami (Figure 5).

### 3.2. TBV and ICV Analysis

The sex and age-related differences seen in the adjustment parameters were analyzed using the moderated regression models. TBV and ICV were subjected to a General Linear Model with a sigma-restricted parameterization as the dependent variables with two covariates (age and sex) and the interaction term. The GLM modeling results are presented in Table 3—they show that TBV and ICV are sex-dependent. Moreover, TBV has a statistically significant negative correlation with age. The sex predictor is dominant in this model and indicates the higher total brain volumes as well as the intracranial volumes in the males (Table 3, Figure 1).

**Table 3:** The General Linear Models with Sigma-restricted parametrization results with two predictors (age, sex) and the age-sex interaction.

	**Age predictor**		**Sex predictor**		**Age × Sex interaction**		**Full model**	
**GLM Models**	β^*^	***p*-value**	β^**^	***p*-value**	β^***^	***p*-value**	***p*-value**
TBV	-0.2416	0.0013	0.8341	0.0011	-0.1696	0.5014	<0.001
ICV	-0.0945	0.2315	0.9658	<0.001	-0.3077	0.2595	<0.001

The results marked in red are statistically significant with p < 0.05.

* The sign (+/-) of β for the age predictor - means the increasing/decreasing brain structure volume with age.

** The sign (+/-) of β for the sex predictor - means the higher/lower brain structure volumes for the males.

*** The sign (+/-) of β for the age-sex interaction - means the increasing/decreasing male/female brain.

*structure volume ratios with age*.

### 3.3. ICV Adjusted GLM Analysis

ICV consistency during aging (Ikram et al., 2012) makes it a reliable tool for correction of head size variation across the subjects in the studies that rely on the morphological features of the brain. It has been utilized as a normalization measure to evaluate age- and sex-related changes in the structures of the brain (Sargolzaei et al., 2015).

Thus, in order to assess whether the sex- and age-related differences of the volumetric measures are significant, the General Linear Models with three covariates, sex, age and ICV, were calculated. The interaction term (age-sex) was also introduced in the calculated models to access if there is a significant difference in the slopes of the trend lines. The results are presented in Table 4. For all except three VOIs (the left hippocampus and the amygdalae) the received models were statistically significant (full model *p* < 0.0146). Whereas for the right hippocampus and both accumbens nuclei the regressor effects (*p*-values) were statistically insignificant. After the ICV adjustment, the sex predictor became statistically insignificant. Generally, the majority of sex differences in the specific VOIs appeared to be linked to the difference in the head size parameter. However, for the white matter volumes the age-sex interaction was observed on the background of the positive correlation with age (Table 4, Figure 6). The age regressor effects revealed to be statistically significant for nine VOIs: gray matter, white matter, CSF, both thalami, both caudate nuclei, left putamen and right pallidum. The regression coefficients for six dependencies show a negative dependence with age. Furthermore, we found the age-dependent increases in the volumes of the white matter, CSF and right pallidum (Table 4, Figure 6).

**Table 4:** The General Linear Models with sigma-restricted parametrization results with three predictors (age, sex, and ICV) and the age-sex interaction.

	**Age predictor**		**Sex predictor**		**ICV predictor**		**Age × Sex interaction**		**Full model**	
**GLM Models**	β^*^	***p*-value**	β^**^	***p*-value**	β^***^	***p*-value**	β^****^	***p*-value**	***p*-value**
GM	-0.4101	<0.001	0.1706	0.2247	0.775	<0.001	-0.1768	0.1878	<0.001
WM	0.2002	<0.001	-0.2420	0.1043	0.8197	<0.001	0.437	0.0025	<0.001
CSF	0.4327	<0.001	0.0266	0.9126	0.8562	<0.001	-0.27	0.2447	<0.001
THA L	-0.2603	<0.001	0.4924	0.067	0.4533	<0.001	-0.2561	0.3204	<0.001
THA R	-0.3237	<0.001	0.2334	0.3368	0.5697	<0.001	-0.0976	0.6734	<0.001
PUT L	-0.2413	0.0046	-0.0318	0.9158	0.4467	<0.001	0.1981	0.4912	<0.001
PUT R	-0.1141	0.1771	-0.1061	0.7272	0.4549	<0.001	0.325	0.2647	<0.001
CAU L	-0.3627	<0.001	-0.6253	0.049	0.5460	<0.001	0.4205	0.1631	<0.001
CAU R	-0.3048	0.0012	-0.6261	0.061	0.5037	<0.001	0.4598	0.1478	<0.001
HIP L	-0.0045	0.966	0.1376	0.7212	0.2474	0.0807	-0.1111	0.7627	0.1263
HIP R	-0.1272	0.214	0.1964	0.5945	0.3097	0.023	-0.1501	0.6698	0.004
AMY L	0.0534	0.6104	0.179	0.6369	0.377	0.0075	-0.3212	0.3757	0.0364
AMY R	-0.0587	0.5774	0.5226	0.1716	0.1203	0.3864	-0.3496	0.3369	0.0487
PAL L	0.1791	0.0461	-0.516	0.1108	0.5996	<0.001	0.5450	0.0781	<0.001
PAL R	0.2453	0.0025	-0.3662	0.202	0.6609	<0.001	0.4533	0.0990	<0.001
ACC L	-0.1923	0.0501	0.0264	0.9401	0.2262	0.0798	0.1921	0.567	<0.001
ACC R	-0.1808	0.0636	0.0594	0.8648	0.1247	0.3285	0.27	0.4187	<0.001

The results marked in red are statistically significant with corrected p < 0.0146.

* The sign (+/-) of β for the age predictor - means the increasing/decreasing brain structure volume with age.

** The sign (+/-) of β for the sex predictor - means the higher/lower brain structure volumes for the males.

*** The sign (+/-) of β for ICV - means the increasing/decreasing brain volume relationship.

**** The sign (+/-) of β for the age-sex interaction - means the increasing/decreasing male/female brain.

*structure volume ratios with age*.

### 3.4. TBV Adjusted GLM Analysis

Total brain volume (TBV) is another measure (to ICV) used to correct the head size variation across subjects. Using TBV may be more appropriate when interest is in how the brain structure volume changes with respect to the brain as a whole. Whereas using ICV may be more appropriate if interest is in how the brain structure volume changes with respect to the maximal adult brain size. Thus, the TBV and ICV correlation generally decrease with age (O'Brien et al., 2011).

In order to adjust the total brain volume in the description of sex and age differences in the analyzed VOIs, the General Linear Models with three covariates (sex, age and TBV) were calculated. Moreover, the age-sex interactions were also included in the received models (Table 5). The results presented in Table 5 show that the amygdalae and the left hippocampus models were statistically insignificant (full model *p* < 0.0146). Moreover, as reveals from the analysis of the structures examined after this adjustment, all sex regressor effects were statistically insignificant.

**Table 5:** The General Linear Models with sigma-restricted parametrization results with three predictors (age, sex and TBV) and the age-sex interaction.

	**Age predictor**		**Sex predictor**		**TBV predictor**		**Age × Sex interaction**		**Full model**	
**GLM Models**	β^*^	***p*-value**	β^**^	***p*-value**	β^***^	***p*-value**	β^****^	***p*-value**	***p*-value**
GM	-0.2643	<0.001	0.1629	0.1171	0.9066	<0.001	-0.2615	0.0095	<0.001
WM	0.3433	<0.001	-0.2115	0.1171	0.9125	<0.001	0.3396	0.0095	<0.001
CSF	0.4893	<0.001	0.3787	0.2211	0.5693	<0.001	-0.4369	0.1413	<0.001
THA L	-0.1609	0.0317	0.4391	0.0847	0.5888	<0.001	-0.2957	0.2236	<0.001
THA R	-0.2093	0.0019	0.2029	0.3666	0.6961	<0.001	-0.1548	0.4719	<0.001
PUT L	-0.1681	0.0574	0.0009	0.9977	0.478	<0.001	0.1418	0.621	<0.001
PUT R	-0.0376	0.67	-0.0793	0.7924	0.4946	<0.001	0.2689	0.3527	<0.001
CAU L	-0.271	0.0037	-0.5926	0.0593	0.593	<0.001	0.3531	0.238	<0.001
CAU R	-0.2188	0.024	-0.6011	0.0686	0.5533	<0.001	0.3987	0.2052	<0.001
HIP L	0.0391	0.7267	0.1453	0.7037	0.2773	0.0694	-0.1402	0.7016	0.1146
HIP R	-0.0727	0.4968	0.2061	0.5725	0.347	0.0182	-0.1866	0.5938	0.0033
AMY L	0.1266	0.2473	0.1675	0.653	0.4503	0.0029	-0.3608	0.3133	0.0175
AMY R	-0.0348	0.7525	0.5175	0.1721	0.1458	0.3305	-0.3619	0.3176	0.0449
PAL L	0.2807	0.0032	-0.4832	0.1305	0.6549	<0.001	0.4716	0.1235	<0.001
PAL R	0.3573	<0.001	-0.33	0.2435	0.7217	<0.001	0.3724	0.1701	<0.001
ACC L	-0.1451	0.1546	0.0081	0.9814	0.2838	0.041	0.1706	0.6072	<0.001
ACC R	-0.139	0.1687	-0.0053	0.9877	0.2219	0.1053	0.2693	0.4136	<0.001

The results marked in red are statistically significant with corrected p < 0.0146.

* The sign (+/-) of β for the age predictor - means the increasing/decreasing brain structure volume with age.

** The sign (+/-) of β for the sex predictor - means the higher/lower brain structure volumes for the males.

*** The sign (+/-) of β for TBV - means the increasing/decreasing brain volume relationship.

**** The sign (+/-) of β for the age-sex interaction - means the increasing/decreasing male/female brain.

*structure volume ratios with age*.

All volumetric parameters with the statistically significant TBV predictor are positively correlated with it (Table 5). However, two statistically significant age-sex interaction terms were obtained for the gray matter and white matter volumes and indicated their steeper volumetric dependence (Figure 7). After the TBV adjustment, the dimorphism in GM and WM is seen and the gray matter volumes seem to be smaller and the white matter volumes are larger with age in males. Furthermore, the volumes of CSF, the left and right pallidum are positively correlated with age, while the volumes of the right thalamus and left caudate nucleus are being reduced with age (Table 5). This dependency is also seen in the scatter plot of the analyzed VOIs (Figure 7).

### 3.5. Influence of the Adjustment Measures on the Volumetric Results

In order to assess the relationship between the two common methods of adjusting for the head (ICV) or brain (TBV) size, we compared the results of both approaches. The aim of this volumetric MRI analysis was to answer the question whether regional atrophy is more predominant than global brain atrophy (Hirabayashi et al., 2016).

When comparing the results of the analysis of covariance (ANCOVA) performed for two types of data adjustment, TBV and ICV, it can be noticed that the age correlations are generally weaker (age regression coefficients) or insignificant for the TBV adjusted models and only for the volumes of white matter, CSF and both pallidi the opposite dependencies are observed (Table 5 vs. Table 4). Importantly, though TBV reflects the global age-related atrophy (Table 3), the TBV adjusted model indicates that the age-related changes of the right THA and left CAU are still significant. Thus, it may be expected that in these anatomical regions, the volume shrinkage is more pronounced than the global atrophy. Moreover, as also seen from this comparison, for the TBV adjusted analysis, all VOIs with the statistically significant adjustment predictors, the adjustment correlation coefficients are higher, with one exception of CSF (Table 5 vs. Table 4). After the ICV adjustment for the gray matter volumes (TBV vs. ICV adjusted GLM), the age-sex interaction becomes insignificant and for the white matter it is stronger (Table 5 vs. Table 4).

## 4. Discussion

With the advent of magnetic resonance imaging, the intracranial volume (ICV) or total brain volume (TBV) have become frequently used as the measures of the brain size to correct for individual variability in the MRI based morphometric studies (Nordenskjöld et al., 2015). In our study, both these parameters in the three covariates GLM models (involving sex, age and the sex-age interaction) were applied. The TBV/ICV adjustment was performed on a group level, and the normalization parameters were included as the covariates in the statistical analysis.

Working with an ethnically homogeneous and healthy population (96 subjects)—not confounded by pathology—provided an excellent opportunity to compare the impacts of both methods of normalization and to study region-to-TBV/ICV dependence inherent to a healthy, but aging, brain. We used one scanner type, the same MRI protocol and performed the radiologist-verified MRI-based volumetric prospective analysis of the imaging data using GLM. Several years ago O'Brien et al. (2006) pointed out that deviations in the regional brain size are sometimes incorrectly assumed in the clinical samples to be related to maldevelopment or pathogenesis—they attributed them to the individual differences in the head, brain, or body size. The authors concluded that the quantitative approaches concerning the comparative use of various adjustment methods were necessary—to our knowledge, today such works are still sparse (Hirabayashi et al., 2016). Adjusting for TBV or ICV greatly increases the statistical power of brain morphometry and is especially essential when quantifying the impact of the demographic factors, like age or sex, on various brain morphometric measurements (Barnes et al., 2010; Shang et al., 2018; Aghamohammadi-Sereshki et al., 2019). However, it is worth noting that different head-size correction strategies are not interchangeable and may yield different results (Perlaki et al., 2014). Thus, the question arises: to what extent do these adjustment methods agree or provide the inconsistent results? As reveals from our results (Table 3, Figure 1), the head size adjustment parameters are significantly larger in men, and the choice of the total head/brain normalization method was found to affect the comparative analyzes with respect to the brain substructures (anatomic regions) (Tables 4, 5). This is due to varying sensitivity of both measures, TBV and ICV, to the processes affecting the brain volume during the lifespan. The brain volume diminishment is claimed to accelerate with age and to be not sex-related, though the latter concerns only the whole brain and not the GM and WM age dependencies (Courchesne et al., 2000; Lemaître et al., 2005; O'Brien et al., 2006; Smith et al., 2007; Barnes et al., 2010; Hirabayashi et al., 2016; Battaglini et al., 2019). In aging populations, it is ICV that tends to reflect better the maximum brain volume reached earlier in life and to normalize the differences in the VOIs sizes (O'Brien et al., 2006, 2011). Both measures were, however, applied by Hirabayashi et al. to describe the hippocampal atrophy in diabetes. Their idea was that such two-way analysis could be helpful in assessing whether the regional atrophy is predominant or the global brain loss is the main loss process in this disease (Hirabayashi et al., 2016). Such an approach makes it, thus, also possible to visualize the local volume loss occurring within the brain substructures with age. Using the ICV measure, though independent on the brain volume loss, introduces an uncertainty from adding to the whole brain volume also a fraction of the CSF volume. The ICV estimation errors associated with the misclassified CSF volumes are reported in some works as leading to overestimating ICV mainly for the females (Nordenskjöld et al., 2015). That is why, in order to improve the classification results (Lindig et al., 2018) and to ensure the optimal segmentation quality, we applied the multimodal segmentation procedure. The results of the GLM analyses using the ICV adjustment (Table 4, Figure 6) indicate the age-related dimorphism of the white matter volumes. Thus, it may be expected that the processes involving the WM are more pronounced for the male group than for the females one. Ge Y et al. described the adjusted WM volume changes during the lifespan—according to their observations, the WM volume increases approximately up to age 40, then decreases (Ge et al., 2002). Farokhian et al. explained that it could be due to the ongoing maturation of the white matter during normal aging (Farokhian et al., 2017). Coming back to our results, it is worth noting that with the TBV adjustment the negative age dependence of the GM volume is more pronounced in the males, while in case of WM the age-related positive trend in the female group is weaker (Table 5, Figure 7). The higher content of gray matter in the female brains after the adjustment for TBV is also reported elsewhere (Gur et al., 1999; Goldstein et al., 2001). Without the head size adjustment, the volumetric data reveal much stronger sex-related influences (Table 2, Figure 5). Thus, the adjustment parameters play a crucial role in linking sex with brain size. Often, the volumetric differences are being explained as resulting from the biological (genes and hormones), environmental influences on the brain development (McCarthy and Arnold, 2011) and the presence/absence of specific habits (i.e., smoking, alcohol consumption, etc.) and/or comorbidities (i.e., hypertension, diabetes, obesity, etc.), particularly with increasing age (De Stefano et al., 2017; Battaglini et al., 2019). On the other hand, it is commonly reported that the larger body size in males results in the larger crania, higher proportion of white matter, and more significant cerebrospinal fluid volume (CSF) (Ritchie et al., 2018). This is also observed in the developing brain (i.e., during childhood and adolescence) (Cosgrove et al., 2007), including subcortical structures (e.g., hippocampus, amygdala and corpus callosum (CC)) (Allen et al., 1991; Giedd et al., 1996, 1997). The changes reported by us in GM and WM for the TBV adjustment seem to indicate that the male brain ages faster than the female one. It is in agreement with the observations by Kiraly et al.—they demonstrated the age and sex dependencies of the subcortical volumes and interpreted them in terms of the faster aging of the males (Király et al., 2016). In a large population of 949 youths Ingalhalikar et al. (2014) found that for the females the relative GM volumes are larger than in the males, whereas the WM volumes are larger in the males. These differences were observed both in the children and adults (Benavides et al., 2019). Wierenga et al. (2018) reported, in turn, a significantly higher variance for several brain structures among the males. Importantly, the histological analyses confirmed the MRI-based observations by showing the increased neuronal densities in the posterior temporal cortex in the females (Witelson et al., 1995). A meta-analysis of the sex differences in the overall and regional brain volumes and the regional brain tissue densities showed not only the larger brain volumes in the males but also the significant sex-related differences in the amygdala, hippocampus, planum temporale and insula (Ruigrok et al., 2014). These authors noted, however, that though the overall volume analyses were all performed on the absolute brain volumes, they were not adjusted for the body weight or height, and such correction factor may also be necessary. We found that the thalami volumes are significantly greater in males, but only when the volumetric data are not related to the head size parameter (Table 2, Figures 3, 5). After such adjustment, this region becomes statistically insignificant (Table 4, Figure 6). However, there are contradictory reports for this structure—no significant sex-related difference was found by Ritchie et al. (2018), while Yanpei Wang et al. observed more rapid decrease of the volume of right thalamus in the males than in the females (Wang et al., 2019).

Our volumetric MRI analysis confirmed the significant effect of the aging process on the brain volume (Table 4, Figure 6) and the subcortical regions for the ICV standardization. When relating the compartmental volume measures to ICV, the statistically significant age-dependent negative relationships concerning the GM, THA, PUT (left) and CAU are seen. At the same time, there is a positive correlation of the WM, CSF, and pallidum (right) volumes (Table 4, Figure 6). The age-related smaller values of the GM and WM volumes with the larger CSF volume in the older subgroups were reported by Lemaitre et al. (2012). Good et al. (2001) examined 465 healthy adults and observed the age-related decline in the GM volumes, but not in the global WM ones (except for the local areas). In this study the global CSF volume was also found to be larger in the older subjects. The smaller volumes of both GM and WM in the older subjects were reported by Farokhian et al. (2017) and Schippling et al. (2017). Some studies show a significantly larger volume of the relative WM for the subjects of the middle age vs. childhood, which is followed by the further steady decline of its volume (Taki et al., 2011; Narvacan et al., 2017), while another study reports no significant effect of aging on WM (Taki et al., 2004). Therefore, it is difficult to correlate the age-related changes in the WM volumes from the various studies. Relating the aging process to TBV highlights the dependencies positively correlated with age, whereas for the negative correlations, the dependencies tend to be generally weaker or insignificant (section 3.5). Moreover, it seems that the local atrophies of the left CAU and right THA volumes are more advanced than the global brain atrophy (Table 5 vs. Table 4). Thus, the proper choice of the adjustment parameter seems to exert an important role in the obtained results. Age-related degeneration in the left basal ganglia (i.e., across the caudate nucleus, putamen, pallidum), being stronger in the male group than in the female one, was observed by Xu et al. (2000). Our data show, in turn, that the age-dependent volume loss is statistically significant in both CAU, THA and the left PUT (Table 4). Several *in vivo* imaging studies have attempted to quantify the age-related changes in the whole brain volume, gray matter, white matter, and the CSF compartments, using various imaging techniques (Good et al., 2001; Curiati et al., 2009; Salat et al., 2009; Farokhian et al., 2017). The contradictory results provided by MRI were linked to the discrepancies in the age ranges, analyzed regions, and assignments of the methodological background (Bas-Hoogendam et al., 2017). It may be, thus, concluded, that choosing the optimal methodology of data collection and analysis is essential to avoid an unacceptable bias. It should also be taken into account, that a high inter-individual variability across the population may strongly complicate a proper determination of the volumetric changes of the brain structures (O'Brien et al., 2006, 2011). Thus, in spite of the existence of several statistical methods for adjusting for individual differences in the overall cranial or brain sizes (O'Brien et al., 2006, 2011), there are still critical controversies concerning the applicability of these strategies, the influences of the group characteristics on the obtained results, and, finally, the validity of the conclusions.

The GLM is a popular, robust method and universal methodology that can be applied in a wide variety of applications with many degrees of freedom (O'Brien et al., 2006, 2011). Its versatility allows it to be used where several predictors of various types with complex dependencies are present. However, it may be inconvenient because of the validation of the methodology assumptions (O'Brien et al., 2011). The optimal fitting with the standardized regression coefficients for the statistically significant relations facilitates the interpretation of the results. It is especially useful when complex interactions or curvilinear relationships are present. Our GLM analysis shows that the ICV normalization influences the sex-related variability of the VOIs and, in consequence, change the interpretation of the sex dependencies (compare Table 4 vs. Table 2). When analyzing the reasons of the inconsistencies of the findings reported in various studies, it may be supposed that the differences in the structural image quality, the applied segmentation techniques, and the post-processing methods (Voevodskaya et al., 2014) may be of importance. The MRI ability to distinguish different structures depends on the tissue contrast resolution, which is low for the subcortical structures and high in the frontal brain regions, where the white matter is claimed to be particularly affected by age, as detected with DTI (Head et al., 2004; Salat et al., 2009). Any hardware instabilities may lead to geometric distortions in segmentation of the brain structures (Skorupa et al., 2014; Guadalupe et al., 2017). The accuracy and reproducibility of the current automatic brain segmentation algorithms have been widely tested (Pardoe et al., 2009; Nugent et al., 2013; Velasco-Annis et al., 2018; Goubran et al., 2020). Different imaging protocols, scanner brands and models, subject positioning in the MR scanner, image artifacts, and partial volume averaging were found to reduce the reproducibility of the segmentation methods (Clark et al., 2006; Han et al., 2006; Gronenschild et al., 2012; Velasco-Annis et al., 2018). Therefore, the results based on the non-standardized and non-validated data may be uncertain. This is very important, especially in case of any subtle volumetric differences that may be masked by technical issues. We tried to avoid such effects by designing prospective investigation with the image acquisition parameters standardized for the entire group and using the same scanner. Additionally, the MRI imaging was done under the experienced radiologist supervision (the same in all examinations), and the volumetric parameters were calculated and analyzed in a native space of the original data. The last stage of the analysis—the classification—was also validated by a visual check of the segmentation results.

The main limitation of the study appears to be medium (<100) sample size. However, the recent papers show (Ruigrok et al., 2014; Tan et al., 2016) that the numbers of the subjects involved in the volumetric analyses in those studies were comparable or smaller (median = 99; Ruigrok et al., 2014; median = 48; Tan et al., 2016). Additional limitation is that the study is cross-sectional. The GLM modeling based on a cross-sectional data has to be undertaken with a caution, because the fit may be driven by a sample characteristics at the start (or the end) of the sampling age (Fjell et al., 2010). The heterogeneous effects found in the cross-sectional studies provide only a general estimation of the age-related trajectories, whereas the longitudinal studies seem to represent the individual age-changes in a more stable manner (Raznahan et al., 2011). On the other hand, the main advantage of the study is the ethnically homogeneous, neurologically healthy and radiologically verified studied group—as it facilitates the comparison of the effects of the TBV or ICV standardization on the brain structure measures.

## 5. Conclusions

The GLM analysis revealed that the sex-related differences should be investigated after normalization of the MRI data to avoid unnecessary bias. The choice of the total head/brain normalization method affects the results of the comparative analyzes with respect to the brain substructures. The two-way analysis, using the TBV/ICV adjustment could help assess whether the regional atrophy is predominant over the global brain changes.

The thalamic volumes were found to be significantly greater in the male group, but after the head size adjustment the results become statistically insignificant. On the other hand the gray matter volumes are lower in older men relative to women even after the TBV adjustment.

Brain volumes are increasingly used as clinical indicators. Therefore, a robust and unbiased reference to the normal ranges of the brain structures volumes is necessary to reduce the false decisions caused by misalignment due to the patients' sex or age.

## Data Availability Statement

The datasets generated for this study are available on request to the corresponding author.

## Ethics Statement

The studies involving human participants were reviewed and approved by Bioethics committee at Maria Sklodowska-Curie Memorial Cancer Center and Institute of Oncology Gliwice Branch, Wybrzeze Armii Krajowej 15, 44-101 Gliwice, Poland (Komisja Bioetyczna - Centrum Onkologii, Instytut im. Marii Sklodowskiej-Curie, Oddział w Gliwicach). The participants provided their written informed consent to participate in this study.

## Author Contributions

MK: manuscript preparation, statistical analysis, statistical interpretation, optimization and methodology selection, data processing and calculations, and data validation. DB: manuscript preparation, statistical interpretation, data acquisition, optimization and methodology selection, data processing and calculations, data segmentation, and data validation. KP-M: manuscript preparation, data acquisition, optimization, and methodology selection. KG: manuscript preparation and data acquisition. PW: manuscript preparation, data validation, and radiological description of the data. BK, AM, and AS: manuscript preparation and concept discussion. MS: manuscript preparation and statistical interpretation. BB-B: manuscript preparation, data validation, radiological description of the data, and group selection.

### Conflict of Interest

The authors declare that the research was conducted in the absence of any commercial or financial relationships that could be construed as a potential conflict of interest.
